# Synthesis of boron nitride nanotubes from unprocessed colemanite

**DOI:** 10.3762/bjnano.4.95

**Published:** 2013-12-04

**Authors:** Saban Kalay, Zehra Yilmaz, Mustafa Çulha

**Affiliations:** 1 Department of Genetics and Bioengineering, Yeditepe University, Atasehir, 34755 Istanbul, Turkey

**Keywords:** boron nitride nanotube, chemical vapor deposition, colemanite, synthesis

## Abstract

Colemanite (Ca_2_B_6_O_11_·5H_2_O) is a natural and new precursor material for the synthesis of boron nitride nanotubes (BNNTs). BNNTs have been synthesized from unprocessed colemanite for the first time. The reaction parameters such as time, catalyst type, catalyst amount and temperature were optimized. It was found that the BNNT formation follows the base growth mechanism, which was initiated with a complex of boron nitride (BN) and iron atoms. The obtained BNNTs were characterized by using SEM, TEM, and spectroscopic techniques such as UV–vis, Raman, FTIR and XRD. The BNNTs were randomly oriented and multi-walled with an outer diameter of 10–30 nm and a wall thickness of 5 nm. This novel BNNT synthesis method can be used to obtain high yield, low cost and pure BNNTs.

## Introduction

Colemanite (Ca_2_B_6_O_11_·5H_2_O) is one of the most important compounds of more than 200 different boron ores. All boron ores include boron oxide (B_2_O_3_) at varying percentages in their chemical composition [1]. The colemanite contains 50.8% B_2_O_3_ and is used in many applications including the fibre glass industry, the ceramic industry, the steel industry, boron alloys, and metallurgy. Another use of colemanite is the synthesis of boric acid (H_3_BO_3_) [2]. B_2_O_3_ and H_3_BO_3_ are also used to synthesize boron nitride nanotubes (BNNT)s [3–4].

BNNTs, structural analogoues of carbon nanotube (CNT)s, have superior properties than CNTs due to their robust structure which resists high temperatures and harsh chemical conditions. They also have a high hydrogen storage capacity due to the ionic nature of the B–N bond [5]. In contrast to CNTs, the BNNTs have a constant and wide band-gap of 5.5 eV. Therefore, they are electrical isolators independent from their size or chirality. In recent studies, it has been indicated that the hydrogen storage capacity of BNNTs is two times greater than that of CNTs [6]. It has been theoretically demonstrated that BNNTs can capture ions selectively creating superhydrophobic surfaces [7–8]. Since hexagonal boron nitrides (h-BNs) have a sp^2^ hybridization, the BNNTs can interact with polymers possessing aromatic rings via π-π interaction. Therefore, the BNNT-polymer composites can be prepared from polymeric structures containing aromatic moities to enhance their dispersibility in organic solvents [9–10]. DNA and proteins such as ferritin are used to disperse BNNTs in aqueous solutions for biological applications [11]. The BNNTs are also used to develop nanoscale microfluodic devices [12]. Although there are different conflicting reports about the toxicity of BNNTs, it has been shown in a recent study with human embryonic kidney cells (HEK293), lung epithelial cells (A549), fibroblast cells (3T3-L1), and alveolar macrophages (RAW 264.7) that BNNTs are more toxic than CNTs [13].

The first BNNTs were synthesized by Chopra et al. with the arc-discharge method [14]. Later, the use of chemical vapor deposition (CVD), laser ablation, ball milling, a template-assisted process, and displacement reactions were reported for the synthesis [15–19]. In the template-assisted synthesis, a commonly used method, the BNNTs are synthesized by using CNTs or an aluminum filter as templates [15,17]. The CNTs interact with B_2_O_3_ and NH_3_ gas to replace C atoms with B and N atoms to form tubes which contain C, B and N. Finally, an oxidation process is applied to remove the remaining C atoms from the tube structure [18]. However, removal of C atoms from the structure is not easy and the obtained product is mostly carbon doped BNNTs. CVD is another commonly used method to synthesize BNNTs [19–22]. The CVD method can be used with or without ball milling technique [19–20]. For instance, Yu et al. first milled amorphous boron with NH_3_, then completed the synthesis by using the CVD method at 1200 °C for 8 hours without the utilization of a catalyst. Zhong et al. obtained BNNTs by using ammonium boron powders and ferrocene with the CVD technique under a N_2_ atmosphere at 1450 °C for one hour. Okan et al. reported a method based on CVD to produce BNNTs in the Fe_2_O_3_/MCM-41 complex-catalyst system from boron powder as a starting material at 600 °C for one hour. In another study, Singhal et al*.* obtained BNNTs from the mixture of KBH_4_/NH_4_Cl between 800 °C and 1000 °C in the presence of N_2_ gas with the CVD method. Wang et al. synthesized BNNTs from the obtained initial product by using B_2_O_3_, Mg, and CaB_6_ at 1150 °C for 6 hours with the CVD technique. In addition, it was shown that in the CVD method, temperature could be decreased to 600 °C with the use of a laser-ablation technique [22]. As can be concluded from these studies, CVD is a commonly used technique to synthesize BNNTs.

The BNNT synthesis and growth mechanism depend on the reaction parameters such as substrate, catalyst and temperature but the mechanism has not been elucidated yet. According to the nucleation theory, the formation of a core depends on surface energy, supersaturation, vapor pressure, temperature and binding energy [23–24]. To synthesize unique, high yield and large scale BNNTs, the synthesis mechanism has to be clearly understood. At the moment, two synthesis mechanisms have been suggested: the base growth mechanism and the tip growth mechanisms [19,25–26]. The linear BNNTs are obtained with the base growth mechanism [19]. In the tip growth mechanism, the catalyst is located on the tip of growing BNNTs [26]. Thus, the BNNTs are generally formed in bamboo-like structures. However, the mechanism of the synthesis BNNTs with the CVD method has not been completely clarified yet.

To the best of our knowledge, unprocessed colemanite has not previously been used as a precursor for the BNNT synthesis. In this study, we demonstrate the synthesis of BNNTs from unprocessed colemanite. The reaction parameters such as the amount of colemanite, the type and amount of catalyst, the reaction temperature and duration were studied and optimized. Finally, the formation mechanism of BNNTs was elucidated. The synthesized BNNTs were characterized with several imaging and spectroscopic techniques including SEM, TEM, HRTEM, FTIR, Raman, UV–vis. The impurity of the BNNTs was verified with ICP-MS and XRD. It was found that the metallic impurities could be removed from crude BNNTs by washing in a hot HCl solution. With this method, randomly oriented BNNTs with 10–30 nm size ranges in high yields can be easily synthesized directly from colemanite.

## Results and Discussion

### BNNT synthesis

**Figure 1:**
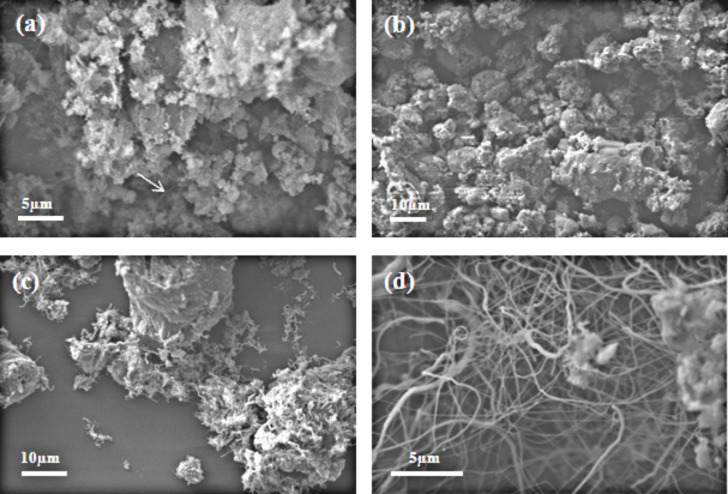
SEM images showing the influence of catalyst on synthesis of BNNTs. (a) No catalyst, (b) Al_2_O_3_, (c) Fe_3_O_4_, and (d) Fe_2_O_3_.

In recent studies, particularly amorphous boron has been preferred as the precursor boron compound to synthesize high-yield BNNTs [5,27–28]. Colemanite as the most important of boron ores can be used for the synthesis of BNNTs since it contains B_2_O_3_ in its structure. In this study, the influence of temperature, type of catalyst, and reaction time on the BNNT yield and the structure were investigated. The most important factor in BNNT synthesis is the proper selection of the catalyst. For this purpose Fe, Al or Mg are widely used in the synthesis of BNNT [3,6]. In this study, four types of catalysts, namely ZnO, Al_2_O_3_, Fe_3_O_4_, and Fe_2_O_3_, were investigated for their performances. Figure 1a–d shows 4 SEM images of reaction mixtures under the same experimental conditions but each of which carried out with a different catalyst. When there is no catalyst, only a few BNNTs are observed in the reaction mixture as seen in Figure 1a (indicated with an arrow). This may be due to the catalysis of Mg or other metal oxide impurities in the colemanite sample. The BNNTs were not formed when ZnO (data not shown) or Al_2_O_3_ (Figure 1b) was used. For the initiation of the synthesis reaction the catalyst in the reaction mixture must be as close as possible to the reaction mixture surface to interact with NH_3_ gas. Since these metal oxides stay buried under the colemanite due to the density difference, they do not effectively interact with NH_3_ gas. Therefore, no BNNT formation was observed. However, when iron oxides were used as catalysts, the formation of BNNTs was dramatically improved. The BNNTs synthesized with the use of Fe_3_O_4_ (Figure 1c) or Fe_2_O_3_ (Figure 1d) are clearly seen in the reaction mixtures on the SEM images. When Fe_3_O_4_ was used, the diameter of the BNNTs was dramatically increased and zigzag structures with shorter length were observed as seen in Figure 1c. When Fe_2_O_3_ was used, a lot of BNNTs with linear but smaller lengths was obtained (Figure 1d). This clearly indicates that the mechanisms of the two different iron oxide catalysts, Fe_3_O_4_ or Fe_2_O_3_, are rather different. The SEM images showed that the BNNTs were obtained in high yield from colemanite as the starting compound with CVD technique in the presence of Fe_2_O_3_ and NH_3_ gas at 1280 °C.

**Figure 2:**
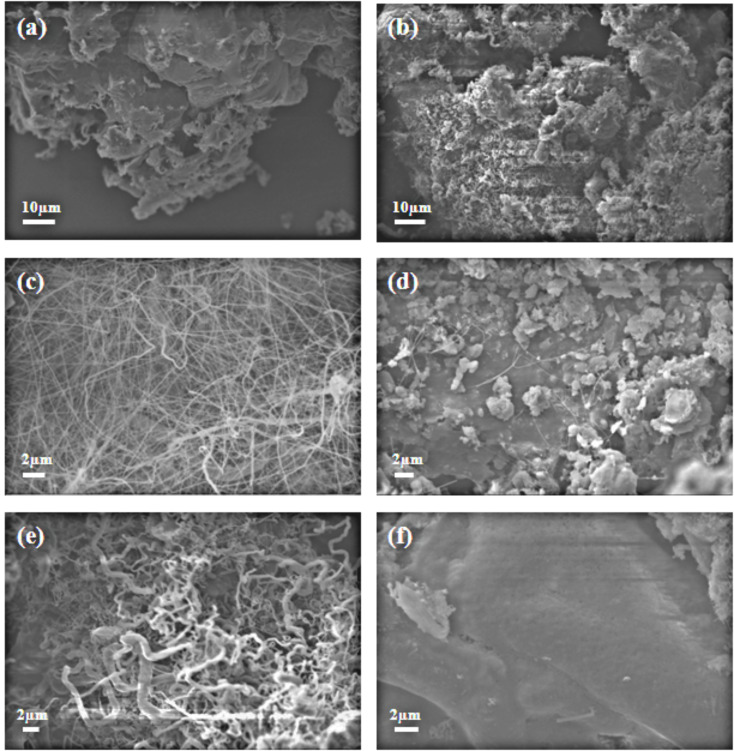
SEM images of the reaction mixtures with increasing reaction times (a–c) and colemanite/catalyst ratios (w/w) (d–f). With a ratio of 12:1 at (a) 30 min, and (b) 60 min. At 120 min with a ratio of (c) 12:1 (d) 32:1 and (e) 8:1. (f) Boat surface after removal of BNNTs, at 120 min and with a ratio of 12:1.

Only the use of Fe_2_O_3_ resulted in high yield BNNTs, so that the reaction conditions in the presence of this catalyst were further optimized. It was found that the optimum temperature for the BNNT synthesis was 1280 °C. Next, the reaction time on the yield and the size composition of BNNTs were investigated. The reaction was set to 30, 60, 120 and 150 min. Then, the obtained BNNTs were analyzed by SEM (Figure 2a–c). No BNNTs synthesis was observed at 30 min. As the reaction time increased from 30 to 120, the formation of BNNTs was more complete. The BNNTs formed at 60 min were shorter. When the time was increased to 120 min, a high yield of the BNNTs was observed. Further increase of the time to 150 min did not alter the yield. It was found that when the reaction time was increased, the length of the formed BNNTs was increased. A mixture of nanotubes and nanowires, whose lengths were around 20 µm, were obtained at 120 min. We concluded that the ideal reaction time for high yield BNNT formation was 120 min.

Next, the influence of the amount of Fe_2_O_3_ on the BNNT formation was investigated. Predetermined 32:1, 16:1, 12:1 and 8:1 colemanite/catalyst (w/w) ratios were tested. A few BNNTs formed when the ratio was 32:1 (Figure 2d). Some BN structures were clustered when the ratio was 16:1 (data not shown). We obtained a rather high yield of BNNTs with a 12:1 ratio of the catalyst (Figure 2c). Finally, an 8:1 ratio of the catalyst caused the formation of BNNTs with a large diameter ,which were thick and had a zigzag structure (Figure 2e). The SEM images presented in Figure 2 show the reaction mixtures obtained with a colemanite/catalyst ratio of 12:1 at 30, 60 and 120 min (a, b and c, respectively), and colemanite/catalyst ratios of 12:1, 32:1 and 8:1 at 120 min (c, d, and e respectively).

**Figure 3:**
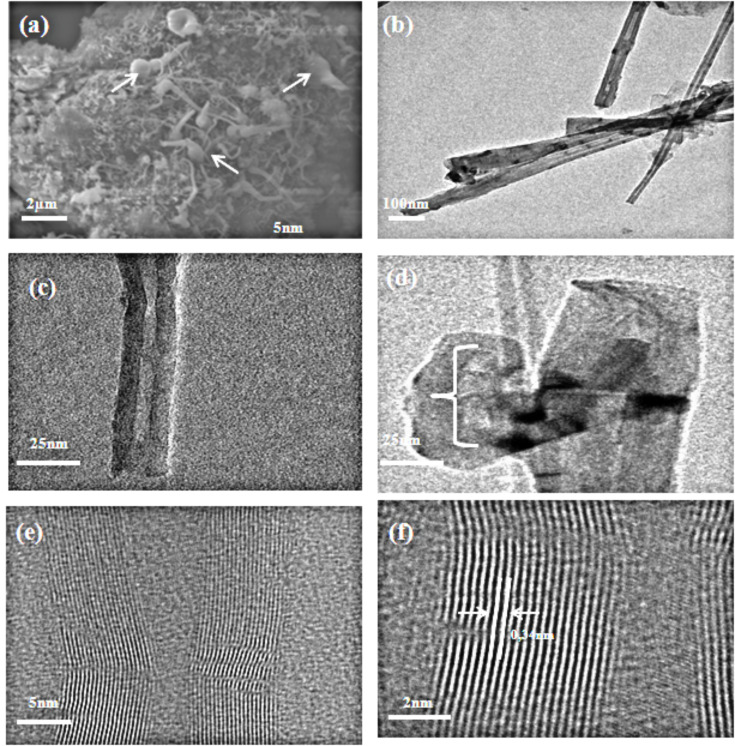
TEM and HRTEM images of BNNTs. (a) SEM image of initial compounds of BNNTs synthesized according to the base growth mechanism (White arrows show initially formed Fe–BN complexes), (b) and (c) TEM image of an open-ended BNNT, (d) TEM image of BNNT with bamboo-like structure, (e) and (f) High resolution TEM images of BNNTs.

Our observations suggest that the BNNT synthesis consists of the clustered structures composed of boron nitride (BN) and iron atoms. The initial BN-Fe complex shown with arrows in Figure 3a has onion-shaped round structures at the bottom. The base growth BNNT formation mechanism proposes that the BNNTs are formed on the surface of the catalysts and the BNNTs grow perpendicular to the catalyst surface as soon as the BN-Fe initial complex is formed [25]. Based on our observations we propose a similar growth mechanism for the formation of BNNTs from colemanite. Another important point in the synthesis of BNNTs is the reaction termination temperature (i.e., the removal of the reaction boat from the tubular furnace). Since a high yield of BNNTs was obtained, the reaction was terminated at 450–550 °C.

The reason for not observing a high yield of BNNTs at temperatures lower than 450 °C may be due to the decomposition temperature of NH_3_, which is 450 °C. The decomposed NH_3_ has an important role in the synthesis of BNNT. It was observed that while the BNNTs were formed on top of the alumina boat, BNNT was not formed at the bottom of the reaction mixture (Figure 2f).

### TEM and HRTEM analysis

The structure of BNNTs was further analyzed with TEM (JEOL-2100). As seen in the TEM images in Figure 3, the BNNTs are multi-walled and have an outer diameter ranging from 10 nm to 30 nm. The Fe_2_O_3_ catalyst was not observed on the tip of BNNTs and they were open-ended (Figure 3b and c). The wall thickness of these BNNTs ranges from 5 nm to 6 nm (Figure 3e and f). The HRTEM image of the synthesized BNNT showed that the distance between the walls was 0.34 nm. We propose that the BNNTs are synthesized according to the base growth mechanism. In this mechanism, metallic Fe in a certain size forms from the Fe_2_O_3_ catalyst. This initial step of metallic catalyst formation is the most important step in the synthesis of BNNTs. When a higher amount of Fe_2_O_3_ is used at the beginning of the synthesis, the formed metallic Fe can form aggregates. When a lower amount of Fe_2_O_3_ is used, a low yield of the BNNT synthesis is observed. This suggests that a critical colemanite/catalyst ratio plays a key role. The second step is the formation of the BN initial complex on the surface of already formed Fe metallic catalyst. This initial complex including metallic Fe catalyst is shown with arrows in Figure 3a. Upon formation of the initial complex, radically decomposed NH_3_ at 1280 °C and boron are involved with the growth on the metallic Fe surface. When B and N are super-saturated, the BN core begins to grow on the surface of the metallic Fe surface. The open-ended BNNTs get longer with the support of continuously added B and N on the surface of the catalyst, and then the BNNTs formation was completed (Figure 3b).

In most of the synthesis where amorphous boron is used as a substrate, it is possible to observe bamboo-like tubular structures, and we detected similar structures in our study [29] (Figure 3d). The formation of metallic Fe clusters with certain sizes also suggests that the BNNT synthesis starts on the metallic Fe cluster surfaces.

Reaction stoichiometry and temperature may result in yielding BNNTs with different morphologies such as open-ended or closed-ended, straight-oriented or randomly-oriented and flower-like with the CVD method. It was reported that the flower-like morphology is only obtainable at 1400 °C [25]. In our study, a few flower-like BNNTs were also observed at 1280 °C.

### Purification of BNNTs, ICP-MS and XRD results

The purification of the synthesized BNNTs was evaluated with ICP-MS. The results showed that the colemanite contains 129.30 ± 1.70 ppm B, 9.10 ± 0.10 ppm Mg, 207.60 ± 4.20 ppm Ca, 5.62 ± 0.08 ppm Sr (see Table 1). In this BNNT crude product synthesized by using colemanite, 40.04 ± 1.66 ppm Fe was found in addition to Ca, B and Mg. The crude product was incubated and stirred in 4 M HCl for four hours at room temperature to remove Fe impurities. Washing with 4 M HCl reduced the Fe and Ca content approximately to a rate of 2/3. When the same washing was applied at 90 °C, Fe, Ca and other impurities were almost completely removed. Note that washing with hot 4 M HCl was very important to obtain pure BNNTs. After washing with HCl solution, pure BNNTs were obtained by washing with 1 M HNO_3_ for six hours. The only observed impurity in the final purified BNNT sample was Ca in an amount of 5.80 ± 0.08 ppm.

**Table 1:** ICP-MS elemental analysis of colemanite, crude and purified BNNTs.

	^11^B ppm	^23^Na ppm	^24^Mg ppm	^27^Al ppm	^48^Ca ppm	^56^Fe ppm	^88^Sr ppm

Control	2.56 ± 0.18	—	—	—	0.74 ± 0.02	—	—
Colemanite	129.30 ± 1.70	0.22 ± 0.02	9.10 ± 0.10	0.34 ± 0.02	207.60 ± 4.20	0.24 ± 0.02	5.62 ± 0.08
Crude Product	34.88 ± 0.20	0.10 ± 0.02	10.20 ± 0.06	0.68 ± 0.02	214.5 ± 8.34	40.04 ± 1.66	5.88 ± 0.02
Pure BNNT	—	—	0.14 ± 0.02	0.16 ± 0.02	5.80 ± 0.08	0.72 ± 0.02	—

**Figure 4:**
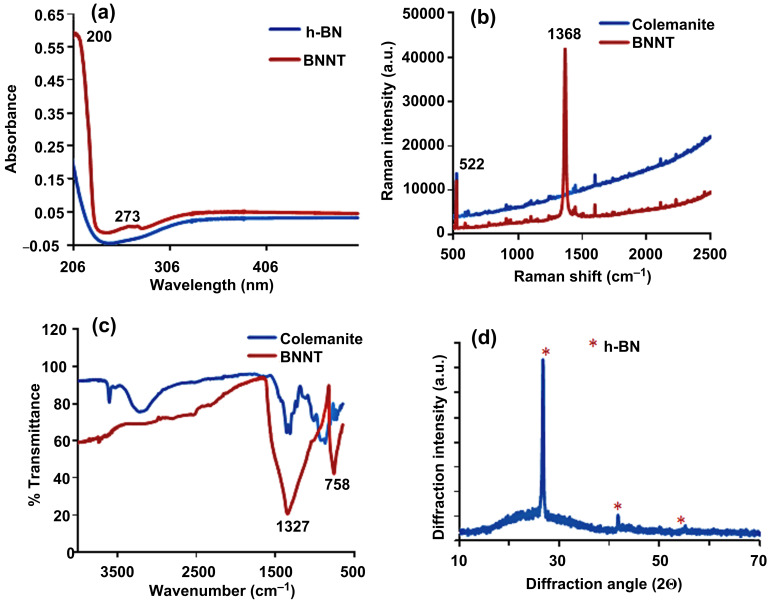
Spectroscopic characterizations of BNNTs: (a) UV spectra of h-BN and BNNTs, (b) Raman spectra of colemanite and BNNTs, (c) FTIR spectra of colemanite and BNNTs, (d) XRD pattern of BNNT.

The XRD pattern obtained from the pure BNNT indicates the presence of a single and dominant h-BN phase (Figure 4d). Peaks were observed at 2θ angles of 26.8° and 41.8° belonging to hexagonal BN. There are not any crystalline phase peaks originating from CaO, SiO_2_, Al_2_O_3_, MgO, SrO or Na_2_O in colemanite and the Fe_2_O_3_ catalyst.

### UV–vis, FTIR and Raman analysis of BNNTs

The BNNTs were analyzed with UV–vis (Figure 4a), FTIR (Figure 4c) and Raman (Figure 4b) spectroscopic techniques. In the UV–vis spectrum of BNNT, a band-gap transition peak at 200 nm was observed. In addition, an absorption peak in the form of a shoulder which is caused by Van Hove singularities was detected at 273 nm [30]. The BNNTs give a sharp peak at 1368 cm^−1^ on the Raman spectrum. This peak shows the E_2g_ in-plane model of h-BN structure and it is due to the bond vibration between B and N, which is located on the same plane [3,21]. E_2g_ vibration peaks were also observed at 1354 cm^−1^ [3,22], 1360 cm^−1^ [21] and 1366 cm^−1^ [25]. The peak observed at 522 cm^−1^ originates from the CaF_2_ slide on the spectrum. Under the same conditions, a vibration peak at 1354 cm^−1^ was not detected on the Raman spectrum of colemanite. The pure BNNTs were also analyzed by FTIR and it was consistent with the reported FTIR spectrum of BNNTs in the literature [20]. The B–N–B in-plane bonding vibration peak at 1327 cm^−1^ and the secondary absorption peak at 758 cm^−1^ were observed. The FTIR spectrum of colemanite has a sharp peak at 3600 cm^−1^, a broad hydroxyl peak at 3200 cm^−1^ due to humidity, and other peaks at 1356 cm^−1^, 1305 cm^−1^ and 886 cm^−1^.

## Conclusions

In this study, BNNTs were synthesized from unprocessed colemanite for the first time by using Fe_2_O_3_ as a catalyst under a NH_3_ atmosphere at 1280 °C and the CDV technique. The findings suggest that for high-yield synthesis of BNNTs the colemanite/catalyst ratio should be 12/1 (w/w). Impurities such as Ca and Fe in the obtained crude product were removed by two hours of washing with a 4 M HCl solution at 90 °C to obtain pure BNNTs. The results of the ICP-MS and the XRD analysis showed that the final obtained BNNTs with an impurity of only 5.8 ppm Ca. It was found that the formation of BNNTs synthesis was based on the radical base growth mechanism, which includes the conversion of Fe_2_O_3_ into metallic iron, the formation of an initial complex between metallic iron and BN, and the growth of BN core into BNNTs on the surface of the metallic iron when the surface was super saturated with B and N atoms. The length of the BNNTs increases as long as the B and N atoms are present in the reaction vessel and stops growing when all boron in colemanite is consumed. The TEM images show that BNNTs formed from colemanite are multi-walled, randomly oriented, have an outer diameter of 10–30 nm and a wall thickness of 5 nm. In conclusion, large scale and pure BNNTs can be obtained by using this novel and simple synthesis method. In addition, the synthesized BNNTs can be purified with a simple acid solution treatment. The obtained BNNTs may be used in many applications including the retention of specific ions, hydrogen storage, and the improvement of the mechanical as well as the chemical durability of polymer composites.

## Experimental

### Material and methods

Colemanite (Ca_2_B_6_O_11_·5H_2_O) was a gift from Eti Mine Works General Management (Turkey). Iron (III) oxide, iron (II, III) oxide, aluminum oxide, zinc oxide, hydrochloric acid, and nitric acid were provided from Sigma. Highly pure NH_3_ gas (99.98%) was provided from Schick GmbH & Co. KG. All solutions were prepared with deionized (DI) water.

#### BNNT synthesis

2 g of colemanite and 0.166 g Fe_2_O_3_ were suspended in 2 mL DI water and vortexed until full dispersion. The homogeneous mixture was transferred into an alumina boat. The water was evaporated with pre-heating at 180 °C for 15 min, and the alumina boat was placed into the center of the tubular furnace (Protherm, Furnaces PTF 14/50/450). The BNNT synthesis was performed under NH_3_ atmosphere. The furnace temperature was set to a heating rate of 8 °C/min until 1280 °C and then heated at this temperature for 3 hours. The furnace was left for cooling down to 520 °C, and the reaction boat was removed from the furnace. The BNNTs were collected from the top of alumina boat and kept in a dry environment at room temperature.

#### Purification

The BNNTs were added into 50 mL 4 M HCl for 4 hours at 90 °C by stirring and then precipitated by centrifugation (14000 rpm, 30 min). The obtained product was stirred in 30 mL of 1 M HNO_3_ for 6 hours at 30 °C and then precipitated by centrifugation at 14000 rpm for 30 min. After the centrifugation, the remaining solid was washed with DI water until all acid was removed. The pure BNNTs were dried at 60 °C overnight.

#### X-Ray powder diffractometer

X-ray powder diffraction (XRD Shimadzu XRD-6000, ICDD PDF 4 software) analyses were carried out with Drive Axis Theta-2Theta. The scan range was 2.000–69.980 in continuous scan with a scan rate of 2.0000 deg/min. The sampling pitch was set to 0.0200 deg, the preset time was 0.60 s.

#### Inductively coupled plasma-mass spectrometer

The analyses were performed by using an X Series 2 Inductively Coupled Plasma-Mass Spectrometer (ICP-MS) (Thermo Scientific) and a CETAC asx-520 auto sampler. Plasma power (1350 W), plasma gas (Ar), Nebulizer gas flow rate (0.95 L/min), uptake time (35 min) and wash time (35 min) were optimized in an ICP-MS system. VHG Labs Z frequency 1007-100 multi-element standard solutions of Al, B, Cu, Ag, As, Cd, Fe, Ni, Sr, Zn, Ca, P, Hg (1000 µg/mL) were used as stock solution. 0.1 µg/mL, 1 µg/mL, 10 µg/mL, 100 µg/mL of standard solution in 5% HNO_3_ from the stock solution were prepared. Each sample was measured for three times and calibration curves were created for each metal. A 10 mg sample was boiled in 5 mL HNO_3_:HCl (2:1 v/v) for 15 min and sonicated for 10 min, and the final volume was completed to 20 mL with DI water. After the sample was dissolved, it was filtered with a 0.2 µm syringe driven filter. The prepared sample was analyzed by ICP-MS.

#### Scanning electron microscopy

The sample was placed on a carbon disc and coated with a few nm thick gold-layers by using a Baltec SDC 005 sputter-coater. Scanning electron microscopy (SEM) images were obtained by using a Carl Zeis Evo-40 instrument under high vacuum with an accelerating voltage of 10 kV.

#### Raman spectroscopy

All Raman Spectroscopic measurements were performed by a completely automated Renishaw InVia Reflex Raman Microscopy system (Renishaw Plc., New Mills, Wotton-under-Edge, UK) equipped with a 514 nm Ar lasers. The laser power was set at 30 mW, and the exposure time was 10 s. A 50× objective was used. The wavelength of the instrument was automatically calibrated by using an internal silicon wafer, and the spectrum was centered at 520 cm^−1^. All spectra were acquired with a 514 nm laser.

#### Transmission electron microscopy

Transmission electron microscopy (TEM) measurements were performed with JEOL-2100. High Transmission electron microscopy (HRTEM) operating at 120 kV (LaB6 filament) equipped with an Oxford Instruments 6498 EDS system.
